# Lung Surfactant for Pulmonary Barrier Restoration in Patients With COVID-19 Pneumonia

**DOI:** 10.3389/fmed.2020.00254

**Published:** 2020-05-22

**Authors:** Ursula Mirastschijski, Rolf Dembinski, Kathrin Maedler

**Affiliations:** ^1^Center for Biomolecular Interactions Bremen, University of Bremen, Bremen, Germany; ^2^Department of Intensive Care and Emergency Medicine, Klinikum Bremen-Mitte, Bremen, Germany

**Keywords:** COVID-19, SARS-CoV-2, pneumonia, lung surfactant, ARDS, inflammation

## Introduction

Corona virus SARS-CoV-2 has already spread around the whole world and is currently, with no vaccine available yet, unstoppable. As per today, COVID-19 affects more than 3,000,000 confirmed patients globally. First line medications are antiviral drugs and multiple urgent clinical trials are under way. However, a recent clinical trial testing the HIV protease inhibitor combination lopinavir and ritonavir showed no significant antiviral activity against SARS-CoV-2 in patients with severe disease (1). As long as we do not have specific antiviral therapies against SARS-CoV-2, we need to provide supportive symptomatic therapies to prevent pulmonary failure, the most common cause of COVID-19 mortality.

## Type II Alveolar Cells Are Damaged by SARS-COV-2

Viral infection and resulting alveolar cell destruction attract immune cells with an excessive alveolar exudative and interstitial inflammatory reaction. A storm of cytokine and chemokine production results in lung tissue destruction and ultimately in severe acute respiratory distress syndrome (ARDS). SARS-CoV-2 as well as SARS-CoV enter the cells through the angiotensin converting enzyme receptor 2 (ACE2). ACE2 is highly expressed on the apical surface of the airway epithelia, vascular endothelia, renal, and cardiovascular tissue as well as various other cells (2). As they enter through the respiratory tract, SARS-CoV, and SARS-CoV-2 may specifically destroy cells, which predominantly express the ACE2 receptor on their surfaces, namely the type II alveolar cells (2, 3).

As progenitor cells for the alveolar epithelium, type II alveolar cells are the “defender of the alveolus” (4). They maintain alveolar homeostasis, especially after microbial lung damage, where they control the inflammatory response.

**Figure 1:**
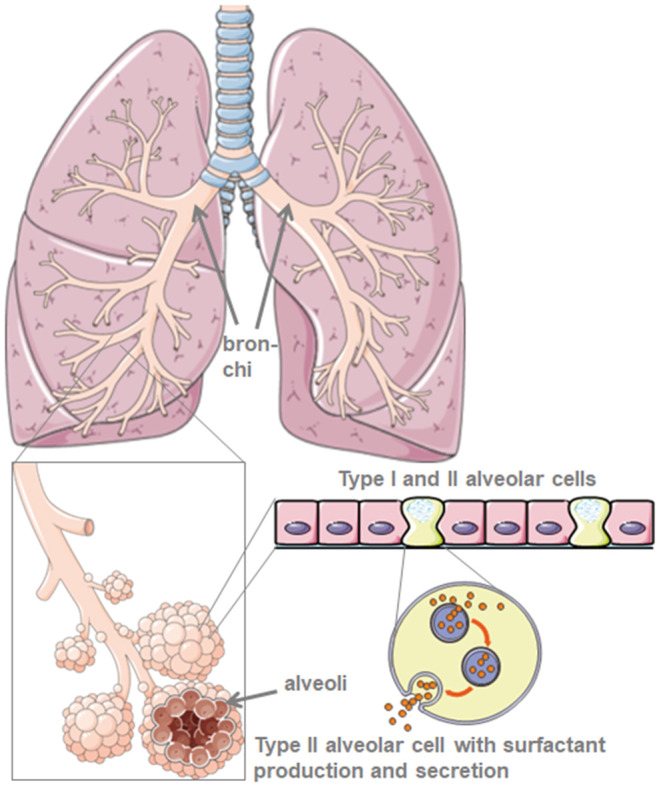
Model of lung and alveolar morphology. Lung surfactant is produced by type-II-alveolar cells. Created using smart servier medical art under https://creativecommons.org/licenses/by/3.0/.

Through their production of the protective lung surfactant, type II alveolar cells reduce the lung surface tension and thus facilitate breathing and gas exchange, and in addition, are central for repair processes after trauma (5) (Figure 1). Damage to type II alveolar cells drastically reduces pulmonary surfactant production and secretion to the alveolar space. This is followed by atelectasis due to lung surfactant dysfunction that further reduces the pulmonary compliance (6). The air-liquid-interphase is perturbed in SARS-CoV-2 infected patients leading to lung damage. ACE2 itself protects from lung injury though anti-inflammatory and anti-fibrotic mechanisms. Thus, the use of recombinant angiotensin converting enzyme (ACE) would not only block virus receptor binding sites but also provide lung protection. In the scenario where SARS-CoV-2 binds to ACE2, protective ACE binding is severely reduced. The destruction of alveolar cells is followed by reduced blood oxygenation, lung fibrosis, oedema, impaired regeneration, and ultimately, leads to respiratory failure (7).

## Lung Surfactant as Protective Anti-Inflammatory ARDS Therapy

Respiratory failure is also known from an entirely different origin, namely in preterm infants with reduced lung surfactant production compared to term-born children. Without sufficient lung surfactant, alveoli collapse during exhalation resulting in poor blood oxygenation.

Lung surfaces depict the air-liquid-interphase and are in constant motion during in- and exhalation. The latter confers the risk of tissue collapse due to fluid surface tension. The lung overcomes this danger by covering its surface with lung surfactant. Lung surfactant is produced in specialized cells found in the terminal lung branches, type II alveolar cells, which start producing lung surfactant immediately after birth (8).

Lung surfactant is a mixture of phospholipids and four surfactant proteins (SP), namely the hydrophilic SP-A and SP-D, also called collectins, and the lipophilic SP-B and SP-C (9). Lung surfactant lowers the surface tension and thereby prevents the alveolar collapse during exhalation. All SP contribute to the innate immune responses of the lung while SP-B and SP-C influence the consistence of the phospholipid rich surfactant as well (10). Recently, novel surfactant associated proteins (SFTA) were described with similar properties compared to the “classic” SP (11–13). SFTA2 is hydrophilic and displays similar properties compared to SP-A and SP-D (13). SFTA3 enhances the phagocytosis of macrophage cell lines (14) and is an amphiphilic protein (12). Therefore, it is likely to be present in the commercially available lipophilic extractions of animal lungs and could enhance the phagocytotic activity of macrophages against CoV-2.

In preterm infants, lung surfactant production is insufficient with poor blood oxygenation and high alveolar surface tension leading to increased inflammatory reaction.

Starting in the late seventies, exogenous bovine, or porcine lung surfactant derived from bronchial lavage was successfully established as a therapy for ARDS in premature infants. Treatment with lung surfactant preparations leads to enhanced oxygenation and increased survival (15–17). Of note, treatment with naturally occurring lung surfactant had a better outcome with regard to infant survival compared to synthetic lung surfactant (17). Natural lung surfactants are a mixture of lipids (90%) and surfactant proteins (10%) which regulate the activity of alveolar macrophages and reduce inflammation. The lipophilic lung surfactant fraction has anti-inflammatory properties when applied intratracheally to the lung (18) as well as topically onto skin (19). In the skin, lung surfactant reduces the expression of pro-inflammatory and pro-fibrotic genes in wounds *in vivo*. In various *in vitro* and *in vivo* murine and human models of wound inflammation, lung surfactant reduced TNF-α, TACE and IL-6 (19), which are highly elevated in severely affected COVID-19 patients.

Recent findings show that SARS-CoV-2 induces the destruction of type II alveolar cells in COVID-19 associated pneumonia (2). Exactly those cells produce lung surfactant and prevent lung collapse. Furthermore, lymphocytopenia with massive release of cytokines is another factor leading to pulmonary failure and death in severe cases of COVID-19 patients. Therefore, anti-inflammatory targets such as anti-TNF and anti-IL-6 have been suggested to better control severe COVID-19 infection (20).

## Discussion: the Use of Lung Surfactant for Pulmonary Barrier Restoration in Patients With COVID-19 Pneumonia

Although lung surfactant therapy is the standard, very safe and effective therapy for neonates with ARDS, treatment with recombinant SP-C based surfactant did not show improved survival in major randomized controlled trials in adults (18). Importantly, the use of natural surfactants seems to be advantageous compared to synthetic surfactants (16, 17) with significant improvement in blood oxygenation and shorter ventilation time in infants (16). Meconium aspiration syndrome resembles COVID-19 pneumonia with reduced surfactant production and destruction of type II alveolar cells (21). Early administration of natural lung surfactant decreased ECMO therapy and ventilation time (21). This suggests that early administration of natural lung surfactant could indeed improve the pulmonary function also in adult patients with severe ARDS, while the cause of death may not be the collapsed lung alone but rather a multi-organ failure. Besides, different risk factors for the development of ARDS and different phenotypes imply possible varying effects due to therapeutic measures. Thus, beneficial effects of surfactant therapy in COVID-19 associated ARDS patients are conceivable, especially when applied early in the treatment strategy against pulmonary failure.

Because of the robust anti-inflammatory and lung protective efficacy and the today's urgent need for lung supportive therapy, we propose the adjuvant treatment of COVID-19 pneumonia patients on ICUs with natural lung surfactants in addition to the current standard of ARDS intensive care treatment. Current evidence suggests that this would increase blood oxygenation, reduce pulmonary oedema, and ameliorate the excessive inflammatory reaction found in lung autopsies of COVID-19 patients (22). Windtree therapeutics™ announced their plan to test KL4, a synthetic surfactant, in severe COVID-19 infected patients (https://www.windtreetx.com/). In Germany, Lyomark Pharma GmbH are planning to test their natural multicomponent lung surfactant bovactant in adult COVID-19 patients with pneumonia as well (www.lyomark.com).

Commercially available lung surfactant is relatively inexpensive for ICU standards, easily available and has no known side effects in children and adults. Caution should be taken in patients with known allergies against bovine or porcine products, as lung surfactants are mostly harvested from bovine (bovactant, Alveofact®) or porcine (poractant alfa, Curosurf®) lungs by lavage or tissue mincing followed by extraction of the lipid fraction.

**Figure 2:**
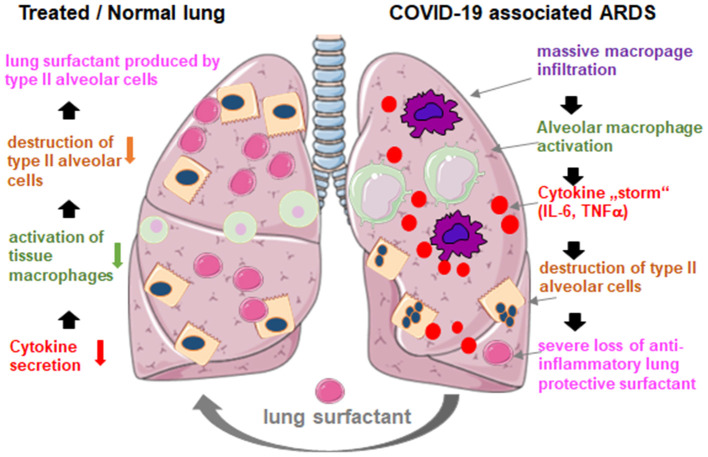
Hypothetical mechanism of externally applied lung surfactant for pulmonary protection in severe COVID-19 associated ARDS. COVID-19 associated ARDS is characterized by massive macrophage infiltration, tissue alveolar macrophage activation and a potentiation of cytokine production in the lung (cytokine “storm”), which leads to the destruction of surfactant producing type II alveolar cells, which worsens the situation through the loss of anti-inflammatory, anti-fibrotic lung surfactant. Exogenous surfactant may reduce inflammation and thus restore pulmonary survival. Created using smart servier medical art under https://creativecommons.org/licenses/by/3.0/.

Administration is simple by adding the reconstituted lyophilizate into the tracheal tube of the ventilated patient delivering the drug directly to the alveolar space. With regard to bovactant, a nebulizer was recently approved for clinical use in the US by the FDA. By covering the outer surface of alveoli, lung surfactant acts directly on inflammatory cells reducing cytokine production and tissue destruction. Thereby, it restores the pulmonary barrier and thus prevents the lung collapse (Figure 2). Consequently, it will reduce the duration of ventilation therapy, facilitate breathing, and thus contribute to patients' recovery.

## Author Contributions

All authors contributed to the design, writing and conceptualization of the manuscript. UM and KM edited and designed the figures.

## Conflict of Interest

The authors declare that the research was conducted in the absence of any commercial or financial relationships that could be construed as a potential conflict of interest.
